# Female Genital Schistosomiasis and HIV-1 Incidence in Zambian Women: A Retrospective Cohort Study

**DOI:** 10.1093/ofid/ofab349

**Published:** 2021-06-30

**Authors:** Amy S Sturt, Emily L Webb, Comfort R Phiri, Maina Mudenda, Joyce Mapani, Barry Kosloff, Maina Cheeba, Kwame Shanaube, Justin Bwalya, Eyrun F Kjetland, Suzanna C Francis, Paul L A M Corstjens, Govert J van Dam, Lisette van Lieshout, Isaiah Hansingo, Helen Ayles, Richard J Hayes, Amaya L Bustinduy

**Affiliations:** 1 Department of Clinical Research, London School of Hygiene and Tropical Medicine, London, UK; 2 MRC International Statistics and Epidemiology Group, London School of Hygiene and Tropical Medicine, London, UK; 3 Zambart, Lusaka, Zambia; 4 Department of Obstetrics and Gynaecology, Livingstone Central Hospital, Livingstone, Zambia; 5 Department of Infectious Diseases, Oslo University Hospital, Oslo, Norway; 6 Discipline of Public Health, University of KwaZulu-Natal, Discipline of Public Health, Durban, South Africa; 7 Department of Cell and Chemical Biology, Leiden University Medical Center, Leiden, the Netherlands; 8 Department of Parasitology, Leiden University Medical Center, Leiden, the Netherlands

**Keywords:** HIV incidence, female genital schistosomiasis, polymerase chain reaction, PCR, parasite, *Schistosoma haematobium*

## Abstract

**Background:**

Female genital schistosomiasis (FGS) has been associated with prevalent HIV-1. We estimated the incidence of HIV-1 infection in Zambian women with and without FGS.

**Methods:**

Women (aged 18–31, nonpregnant, sexually active) were invited to participate in this study in January–August 2018 at the final follow-up of the HPTN 071 (PopART) Population Cohort. HIV-1-negative participants at enrollment (n = 492) were included in this analysis, with testing to confirm incident HIV-1 performed in HPTN 071 (PopART). The association of incident HIV-1 infection with FGS (*Schistosoma* DNA detected by polymerase chain reaction [PCR] in any genital specimen) was assessed with exact Poisson regression.

**Results:**

Incident HIV-1 infections were observed in 4.1% (20/492) of participants. Women with FGS were twice as likely to seroconvert as women without FGS but with no statistical evidence for a difference (adjusted rate ratio, 2.16; 95% CI, 0.21–12.30; *P *= .33). Exploratory analysis suggested an association with HIV-1 acquisition among women with ≥2 positive genital PCR specimens (rate ratio, 6.02; 95% CI, 0.58–34.96; *P *= .13).

**Conclusions:**

Despite higher HIV seroconversion rates in women with FGS, there was no statistical evidence of association, possibly due to low power. Further longitudinal studies should investigate this association in a setting with higher schistosomiasis endemicity.

In 2019, an estimated 56 million women were living with female genital schistosomiasis (FGS), a neglected tropical disease that results when eggs from the parasite *Schistosoma haematobium* are deposited in reproductive tract tissues [1]. Tissue-entrapped eggs incite a cellular response [2], ultimately resulting in FGS-related morbidity, including infertility [3], and distinct cervicovaginal manifestations [4, 5]. In Sub-Saharan Africa, there is a geographical association between areas of high *S. haematobium* prevalence and HIV-1 infection [6], and FGS has been associated with prevalent HIV-1 [7] with biological plausibility for a causal relationship [3, 8]. Despite global advances in HIV-1 treatment and prevention, gender-related disparities still exist, with particularly heightened risk among young women aged 15–24 years [9]. HIV-1 vulnerability in young women is multifactorial, including biological, behavioral, demographic, social, and structural components [10]. The potential role of FGS as an underreported and preventable co-factor in HIV-1 vulnerability needs further investigation.

Disruption of the protective vaginal and cervical epithelium by FGS-associated lesions may increase HIV-1 susceptibility by providing a portal for viral entry [3, 11]. Additionally, the environment created by *S. haematobium* eggs is more vascular [12], with an increased density of CD4+ lymphocytes [8] compared with non-egg-containing tissue. Thus, tissue-entrapped *S. haematobium* eggs create a cellular milieu that may promote HIV-1 infection. Both *S. haematobium* and *S. mansoni* infection have been associated with prevalent HIV-1 [11]. A cross-sectional study of women with FGS, defined as parasite eggs detected in genital tissue, describes a strong association with prevalent HIV-1 but no evidence of an association between urinary schistosomiasis and HIV-1 [7]. Studies primarily evaluating urinary *S. haematobium* (without universal evaluation for genital involvement) and prevalent HIV-1 have been mixed, with evidence of an association with prevalent HIV-1 in a study of Tanzanian women [13], some evidence of an association with prevalent HIV-1 in a study of Zimbabwean women [14], but with no evidence of an association with prevalent HIV-1 in men and women with urinary *S. haematobium* in Congo [15]. While *S. haematobium* seropositivity in women has been associated with HIV-1 acquisition [16], the association of FGS with incident HIV-1 has not been described.

FGS diagnosis is challenging, and its burden is likely underreported. The presence of parasite eggs or DNA in cervicovaginal tissue is diagnostic of FGS [17, 18], and, historically, biopsy is used as a reference standard [4, 19]. However, theoretical concerns regarding post biopsy HIV-1 acquisition have limited the acceptance of cervical biopsy in research settings [17]. Polymerase chain reaction (PCR) on cervicovaginal lavage (CVL) is a less invasive means of FGS diagnosis, albeit with imperfect sensitivity [17, 18]. Well-defined clinical manifestations have been associated with FGS [5, 20] but are variably correlated with the presence of *S. haematobium* eggs [19, 20] or DNA [17] in genital tissue. The identification of clinical lesions, such as homogenous yellow sandy patches and abnormal blood vessels, with colposcopy is observer-dependent and subject to low specificity [21]. Indeed, homogeneous yellow sandy patches have also been associated with herpes simplex virus–2 and human papillomavirus, and abnormal blood vessels may be associated with cervical intraepithelial neoplasia [5]. Urine microscopy and circulating anodic antigen (CAA) can be used to detect active schistosome infection [18, 22] but do not assess involvement of genital tissue. While either *S. haematobium* and *S. mansoni* can cause FGS, the majority of cases are attributed to *S. haematobium* [23, 24], and the current study focuses on *S. haematobium*. We conducted an array of diagnostic tests for *S. haematobium* infection (CAA and urine microscopy) and FGS (portable colposcopy, cervical swabs, vaginal swabs, and cervicovaginal lavage) and have previously demonstrated that self-collected genital swabs had comparable sensitivity to clinic-based, midwife-collected CVL for the detection of *Schistosoma* DNA by real-time PCR [18].

The longitudinal follow-up of women in the HPTN 071 (PopART) trial in 2 schistosomiasis-endemic communities in Zambia provided an opportunity for a nested study exploring the association of FGS with HIV-1 incidence.

## METHODS

### Study Setting and Participants

The cross-sectional bilharzia and HIV (BILHIV) study was nested in HPTN 071 (PopART), a cluster randomized trial assessing the impact of an HIV-1 combination prevention package including “universal testing and treatment” [25]. HIV-1 incidence was measured in a population cohort (PC) comprised of 1 randomly selected adult (18–44 years of age) from a random sample of households in each community who provided data and blood samples at baseline and at 12, 24, and 36 months [25]. Between January and August 2018, after the 36-month HPTN 071 (PopART) PC visit, trained community workers conducted home visits to women who had expressed interest in the BILHIV study [18]. Women in Livingstone, Zambia, were eligible if they were 18–31 years old, not pregnant, sexually active, and residing in 1 of the 2 urban *S. haematobium*–endemic communities that participated in 1 of 2 HPTN 071 (PopART) intervention arms.

Following written informed consent, the BILHIV study home visit included a questionnaire, genital self-sampling (cervical and vaginal), and a urine specimen, as previously described [18]. Within days of self-sampling, nonmenstruating participants were invited to attend Livingstone Central Hospital for cervicovaginal lavage (CVL) [18]. Cervicovaginal images were captured with a portable colposcope (MobileODT, Tel Aviv, Israel) and evaluated by 1 author (E.F.K.) for the presence of any of the 4 accepted FGS cervicovaginal manifestations: homogenous yellow sandy patches, grainy sandy patches, rubbery papules, and abnormal blood vessels [26]. Women with evidence of schistosome infection by colposcopy [26] or any positive urine or genital diagnostic were treated free of charge with 40 mg/kg of praziquantel. Routine testing for sexually transmitted infections (STIs) was not performed. Participants with suspected STI were offered syndromic management, as per local guidelines [27].

### HIV-1

Laboratory-based fourth-generation HIV-1 testing (Abbott Architect HIV Ag/Ab ComboAssay, Wiesbaden, Germany) was performed for HPTN 071 (PopART) PC participants at each study visit [25]. Additional testing using antigen/antibody screening tests, a discriminatory test, and an HIV-1 RNA test was used to confirm incident HIV-1 infection, as previously described [28].

### Circulating Anodic Antigen

CAA levels reflect the burden of live schistosomes and decline after successful treatment with praziquantel [22, 29]. An up-converting reporter particle lateral flow assay for the quantification of CAA in urine was performed at the Leiden University Medical Center (LUMC), as previously described [18, 30]. Analyzing the equivalent of 417 μL of urine, a CAA value of >0.6 pg/mL was considered positive [22].

### PCR for Detection of Schistosoma DNA

DNA extraction and PCR was performed at LUMC as previously described, using a custom automated liquid handling station (Hamilton, Switzerland) [20, 31]. DNA was extracted from 200 μL of specimen (cervical swab, vaginal swab, CVL) with QIAamp spin columns (QIAGEN Benelux, Venlo, the Netherlands). Detection of the schistosome-specific internal-transcribed-spacer-2 (ITS2) target was performed by real-time PCR as previously described [18, 31]. This PCR does not differentiate between *Schistosoma* species. DNA amplification and detection were performed with the CFX96 Real Time PCR Detection System (BioRad, Hercules, CA, USA). The output in threshold cycles (Cts), reflecting the parasite-specific DNA load in the tested sample, was analyzed using BioRad CFX software. Parasite DNA loads were categorized by the following prespecified C_t_ thresholds: high (C_t_ < 30), moderate (30 ≤ C_t_ < 35), low (35 ≤ C_t_ < 50), and negative (no C_t_ detected), as previously described [32].

### Patient Consent

This study was approved by the University of Zambia Biomedical Research Ethics Committee (reference 011-08-17), the Zambia National Health Research Authority, and the London School of Hygiene and Tropical Medicine Ethics Committee (reference 14506). Permission to conduct the study was given by the Livingstone District Health Office and the Livingstone Central Hospital superintendent. Each participant provided written informed consent.

### FGS Definitions

**Figure 1.**
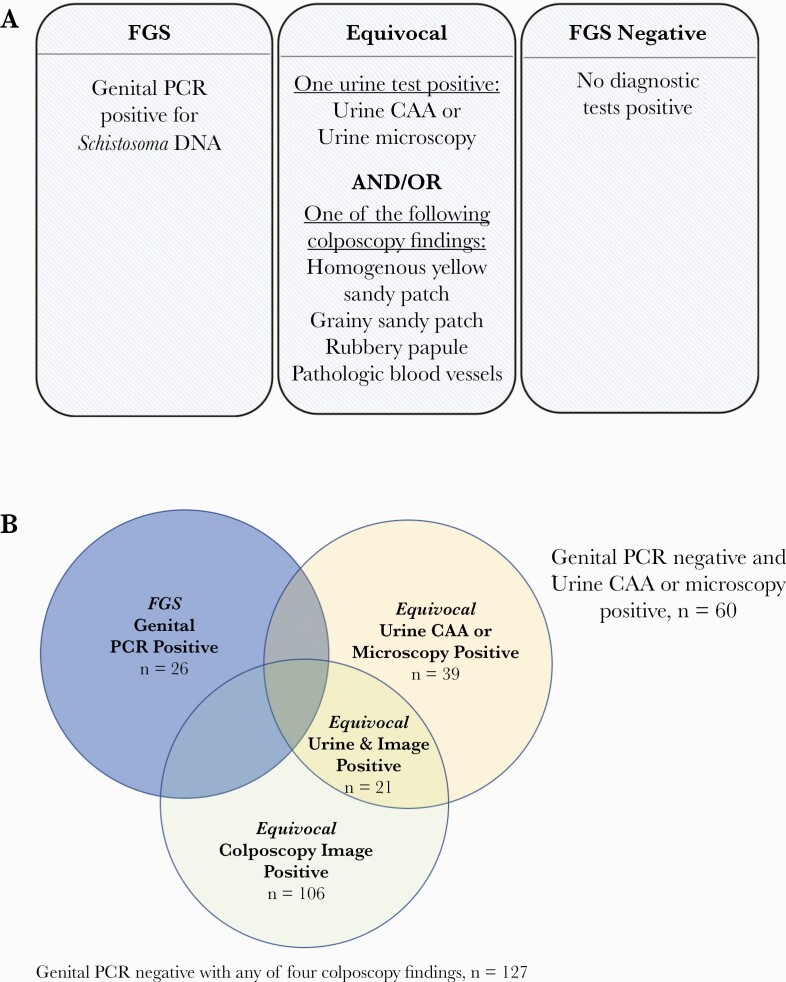
Female genital schistosomiasis categories and Venn diagram illustrating results by diagnostic test type. A, Female genital schistosomiasis diagnostic categories. B, Participants in the diagnostic categories by test result. Participants within the FGS and probable/possible FGS categories do not overlap. Abbreviations: CAA, circulating anodic antigen; FGS, female genital schistosomiasis; PCR, polymerase chain reaction.

Comparison groups were defined by the results of 4 investigations: genital PCR, colposcopy image review, urine CAA, and urine microscopy. Participants were grouped by the outcomes of their diagnostic tests into 3 mutually exclusive categories. *FGS* was defined as at least 1 positive genital PCR (cervical swab, vaginal swab, or CVL) (Figure 1). *Probable/possible FGS* was defined as the presence of either a positive urine diagnostic (CAA or microscopy) or 1 of 4 cervicovaginal manifestations suggestive of FGS on portable colposcopy, or both, with negative genital PCR (Figure 1). FGS *negative* was defined as negative results on all diagnostics.

### Statistical Methods

Characteristics of study participants were summarized by frequency and percentage. Women with HIV-1 (WWH) at HPTN 071 (PopART) baseline were excluded from further analyses. HIV-1 incidence was calculated as the number of seroconversions per 1000 person-years of follow-up. Participants contributed person-time for the calculation of HIV-1 incidence starting with their first HIV-1 test and ending at the date of HIV-1 seroconversion for those who seroconverted, or at the date of last follow-up or the end of scheduled follow-up (whichever occurred earliest) for women who did not seroconvert. HIV-1 seroconversion was assumed to occur at the midpoint between the last negative and the first positive HIV-1 test. We assumed that FGS acquisition occurred before HPTN 071 (PopART) enrollment [33]. BILHIV study participants were consecutively recruited from the PC, providing the opportunity to compare the rate of incident HIV-1 infection in women with and without FGS, with power determined by the number of HIV-1 seroconversions and FGS prevalence. Data on HIV-1 outcomes were not available until after BILHIV study closure.

Associations of risk factors with incident HIV-1 infection were calculated as rate ratios and 95% confidence intervals, estimated using exact Poisson regression in univariable and multivariable analysis. We used a causal conceptual framework to inform our choice of potential confounders. A priori, we included age as a confounding variable. Due to loss of precision with further adjustment for potential confounding variables, no additional parameters were included in the multivariable model. To assess the primary exposure of interest, women with FGS (n = 26) were compared with an FGS-negative comparison group comprising those who were negative on all diagnostic investigations (n = 218). Participants who were negative on all diagnostic investigations but missing colposcopy images (n = 82) were excluded from the primary analysis.

To evaluate the association of schistosome infection intensity with HIV-1 seroconversion, 2 ad hoc exploratory analyses were performed. One compared participants with FGS and a moderate/high *Schistosoma* DNA concentration (Ct < 35) with those in the FGS-negative group. The second compared participants with FGS with ≥2 positive genital PCR specimens with those in the FGS-negative group. Data were analyzed using STATA 15.1 (Stata Corporation, College Station, TX, USA).

## RESULTS

**Figure 2.**
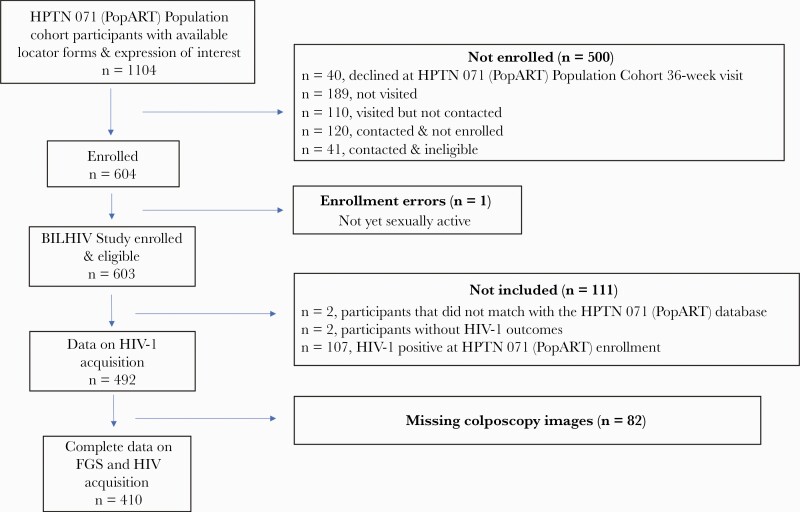
Study flow diagram. Not visited (n = 189): the participant was not visited before the study closed for enrollment. Visited but not contacted (n = 110): a visit was made to the study household, but the participant could not be located (70), had relocated (39), or died (1). Contacted & not immediately enrolled (n = 120): visited but not recruited (42), out of town (18), declined to participate (60). Contacted & ineligible (n = 41): virgin (16), pregnant (17), over 31 (8). Abbreviation: FGS, female genital schistosomiasis.

A total of 603 eligible women from the HPTN 071 Population Cohort were enrolled in the BILHIV study. WWH at HPTN 071 (PopART) trial entry (n = 107, 17.7%) were excluded, with 492 (82.1%) included in this analysis (Figure 2). Of the included participants, 14% (69/492) did not attend clinic for CVL.

### Baseline Characteristics

The majority of participants had received at least secondary education, were not working, and reported being currently sexually active. A small proportion of women reported current water contact, but more than half reported childhood water contact. Active schistosome infection, defined as either a positive urine microscopy (5.5%, 27/492) or detectable CAA (15.1%, 74/492), was detected in 15.7% (77/492) of participants.

### HIV Incidence

The 492 women without HIV-1 at HPTN 071 (PopART) study entry provided a total of 1164 person-years of follow-up, during which time 20 (4.1%) incident HIV-1 infections were measured, for an overall rate of 17.2 (95% CI, 11.1–26.6) seroconversions per 1000 person-years. HIV-1 incidence rates are shown by baseline characteristics in Table 1.

**Table 1.** HIV-1 Seroconversion Rates by Baseline BILHIV Study Characteristic in 492 Zambian Women

Sociobehavioral Characteristics		No. (%)	Events	Rate of HIV Seroconversion per 1000 p/y	Crude IRR (95% CI)	*P* Value
Age, y	18–24	289 (58.7)	15	23.6 (14.2–39.2)	Reference	.06
	25–31	203 (41.3)	5	9.5 (3.9–22.7)	0.40 (0.15–1.10)	
Marital status	Single	219 (44.5)	12	23.0 (13.1–40.5)	Reference	.35
	Married or cohabitating	255 (51.8)	7	11.8 (5.6–24.7)	0.51 (0.20–1.30)	
	Widowed, divorced, or separated	18 (3.7)	1	21.2 (3.0–150.6)	0.92 (0.12–7.10)	
Education (highest level)	None or any primary school	137 (27.9)	5	15.7 (6.5–37.6)	Reference	.57
	Any secondary school	297 (60.4)	11	15.5 (8.6–28.0)	0.99 (0.34–2.85)	
	Trade, degree or higher	58 (11.8)	4	29.4 (11.0–78.4)	1.88 (0.50–7.00)	
District	Community A	260 (52.9)	11	15.5 (8.6–28.0)	Reference	.60
	Community B	232 (47.2)	9	19.8 (10.3–38.0)	1.27 (0.53–3.07)	
Household members	1–3	141 (28.7)	12	38.4 (21.8–67.6)	Reference	.007^a^
	4–5	201 (40.9)	4	8.2 (3.1–21.7)	0.21 (0.07–0.66)	
	6+	150 (30.5)	4	11.1 (4.2–29.5)	0.29 (0.09–0.90)	
Employment status	Not working	327 (66.5)	13	17.1 (10.0–29.5)	Reference	.99
	Working	165 (33.5)	7	17.3 (8.2–36.2)	1.01 (0.40–2.52)	
Sexual behavior characteristics						
Age at sexual debut, y	8–16	197 (40.0)	9	20.6 (10.7–39.5)	Reference	.79
	17–19	220 (44.7)	8	14.9 (7.5–29.9)	0.73 (0.28–1.88)	
	20–24	75 (15.2)	3	15.7 (5.1–48.8)	0.77 (0.21–2.83)	
Lifetime sexual partners	1	149 (30.3)	2	5.7 (1.4–22.9)	Reference	.01^a^
	2	134 (27.2)	5	15.9 (6.6–38.3)	2.78 (0.54–14.34)	
	3	103 (20.9)	5	18.7 (7.8–45.0)	3.27 (0.63–16.85)	
	4+	106 (21.5)	8	34.1 (17.1–68.2)	5.95 (1.26–28.02)	
Currently sexually active^b^^,^^c^	No	63 (12.9)	3	20.1 (6.5–62.4)	Reference	.78
	Yes	427 (87.1)	17	16.9 (10.5–27.1)	0.84 (0.25–2.86)	
STI history^d^	No	466 (94.9)	16	14.4 (8.8–23.5)	Reference	0.009
	Yes	25 (5.1)	4	82.8 (31.1–220.5)	5.76 (1.92–17.22)	
Condom use with last sex^e^	No	367 (75.8)	12	13.8 (7.9–24.3)	Reference	.11
	Yes	117 (24.2)	8	29.2 (14.6–58.3)	2.11 (0.86–5.16)	
Contraceptive use						
Condoms	No	407 (82.7)	14	14.8 (8.8–25.0)	Reference	.23
	Yes	85 (17.3)	6	27.5 (12.4–61.2)	1.86 (0.71–4.83)	
OCP	No	440 (89.4)	18	17.3 (10.9–27.4)	Reference	.96
	Yes	52 (10.6)	2	16.6 (4.1–66.3)	0.96 (0.22–4.14)	
Injectable	No	225 (45.7)	14	22.5 (13.4–38.1)	Reference	.13
	Yes	267 (54.3)	6	11.1 (5.0–24.6)	0.49 (0.19–1.28)	
Implant	No	466 (94.7)	18	16.3 (10.3–25.9)	Reference	.37
	Yes	26 (5.3)	2	34.0 (8.5–135.8)	2.10 (0.48–8.99)	

Abbreviations: BILHIV, cross-sectional bilharzia and HIV study; IRR, incidence rate ratio; OCP, oral contraceptive pill; STI, sexually transmitted infection.

^a^Test for trend *P* value.

^b^Any sexual activity in the last 6 months.

^c^Participants who responded with “no answer” (n = 2) are not shown in the table (HIV seroconversions = 0).

^d^STI history was self-reported; participants who responded with “no answer” (n = 1) are not shown (HIV seroconversions = 0).

^e^Participants who responded with “no answer” (n = 8) are not shown in the table (HIV seroconversions = 0).

HIV-1 incidence rates were 23.6 (14.2–39.2) in women aged 18–24 years compared with 9.5 (3.9–22.7) in women aged 25–31 (rate ratio [RR], 0.40; 95% CI, 0.15–1.10; *P* = .06) (Table 1)). The HIV-1 seroconversion rate decreased as the household size increased (*P *= .007, test for trend) and increased as the number of lifetime sexual partners increased (*P *= .01, test for trend). Women self-reporting a history of STI were more likely to seroconvert than women without self-reported STI (RR, 5.76; 95% CI, 1.92–17.22; *P *= .009) (Table 1). No other sociodemographic or behavioral characteristics were associated with HIV-1 incidence. After adjusting for age, there remained strong evidence that a lower number of people residing in a household (*P *= .008, test for trend), a higher number of lifetime sexual partners (*P *= .01, test for trend), and self-reported history of STI (aRR, 6.05; 95% CI, 2.02–18.12; *P *= .008) were associated with HIV-1 seroconversion. Additionally, there was no evidence of an association between urinary schistosome infection (as defined by urine CAA and/or microscopy) and HIV-1 seroconversion (Table 2).

**Table 2.** HIV-1 Incidence by FGS Status and Schistosomiasis-Related Factors

Category	No. (%)	Incident HIV Cases	Total PY	Rate per 1000 PY (95% CI)	IRR (95% CI)	*P* Value	aRR^a^	*P* Value
FGS negative^b^	218 (53.2)	6	532.0	11.3 (5.1–25.1)	Reference	.26^c^	Reference	.33^c^
Probable/possible FGS	166 (40.5)	7	372.2	18.8 (9.0–39.5)	1.67 (0.48–6.01)		1.73 (0.50–6.22)	
FGS	26 (6.3)	2	64.5	31.0 (7.8–123.9)	2.75 (0.27–15.36)		2.16 (0.21–12.30)	
Exploratory analysis of participants with FGS								
FGS negative	218	6	532.0	11.3 (5.1–25.1)	Reference	.09	Reference	.13
FGS and 2-3 PCR positive^d^	13	2	24.9	80.4 (20.1–321.7)	7.13 (0.70–39.89)		6.02 (0.58–34.96)	
FGS negative	218	6	532.0	11.3 (5.1–25.1)	Reference	.15	Reference	.19
FGS and PCR Ct <35^e^	13	2	32.6	61.31 (15.33–245.14)	5.44 (0.54–30.40)		4.73 (0.46–27.05)	
Schistosomiasis-related factors^f^								
Urine microscopy negative	465 (94.5)	18	1102.4	16.3 (10.3–25.9)	Reference	.40	Reference	.47
Urine microscopy positive	27 (5.5)	2	61.6	32.5 (8.1–129.9)	1.98 (0.46–8.58)		1.78 (0.41–7.71)	
Urine CAA^g^ not detectable	416 (84.9)	16	993.5	16.1 (9.9–26.3)	Reference	.86	Reference	.78
Urine CAA detectable	74 (15.1)	3	166.6	18.0 (5.8–55.8)	1.12 (0.33–3.84)		1.19 (0.35–4.10)	
Active infection^g^^,^^h^ not present	413 (84.3)	16	985.0	16.2 (10.0–26.5)	Reference	.93	Reference	.85
Active infection present	77 (15.7)	3	175.1	17.1 (5.5–53.1)	1.05 (0.31–3.62)		1.13 (0.33–3.88)	

Abbreviations: aRR, adjusted rate ratio; CAA, circulating anodic antigen; Ct, cycle threshold; FGS, female genital schistosomiasis; IRR, incidence rate ratio; PCR, polymerase chain reaction; PY, person-years; RR, rate ratio.

^a^Adjusted for age.

^b^Eighty-two participants who were negative on all diagnostic tests but missing portable colposcopy images were excluded from this analysis.

^c^Test for trend *P* value (RR per unit of the exposure variable [FGS negative, probable/possible FGS, and FGS] treated as a continuous variable).

^d^n = 13 excluded (1 genital PCR specimen positive).

^e^n = 13 excluded (Ct >35).

^f^n = 492, unless otherwise specified.

^g^n = 490, 2 vials arrived at the laboratory empty; HIV-1 seroconversion occurred in (n = 1) of these participants.

^h^Defined as detectable urine CAA or positive urine microscopy.

### Association Between FGS and HIV-1 Seroconversion

FGS was identified in 5.3% of women (26/492), defined as any positive genital PCR (cervical swab 3.5% [17/492]; vaginal swab 2.4% [12/492]; or CVL 3.1% [13/423]). Among women with a negative genital PCR, results from both urine and colposcopy imaging were positive in 4.5% (21/466) of participants, and results from either urine or colposcopy imaging were positive in 31.1% (145/466). Of the participants with probable/possible FGS, 63.8% (106/166) had colposcopy changes in isolation (Figure 1), of whom 62.3% (66/106) had abnormal blood vessels and 37.7% (40/106) had grainy or homogenous yellow sandy patches on colposcopy. There were 218 (44.3%) participants who were negative on all diagnostic tests. The rate of HIV-1 seroconversion (per 1000 person/year) in women with FGS (31.0 [7.8–123.9]) was higher than in the FGS-negative group (11.3 [5.1–25.1]) (Table 2) but without statistical evidence of a difference between these rates in either univariable or multivariable analyses (crude RR, 2.75; 95% CI, 0.27–15.36; *P *= .26; aRR, 2.16; 95% CI, 0.21–12.30; *P *= .33) (Table 2).

### Exploratory Analyses: Schistosoma DNA Concentrations and Disease Burden

In the ad hoc exploratory analysis of women (n = 13) with FGS and moderate/high *Schistosoma* DNA concentrations, the IRR for HIV-1 acquisition after adjusting for age was 4.73 (0.46–27.05; *P *= .19) compared with FGS-negative participants (Table 2). In an ad hoc exploratory analysis of women (n = 13) with ≥2 positive genital PCR specimens compared with FGS-negative participants, the IRR for HIV-1 acquisition after adjusting for age was 6.02 (0.58–34.96; *P *= .13) (Table 2). In these groups, n = 9 of the women overlapped and the same 2 participants contributed seroconversions in both groups. There were no HIV-1 seroconversions in participants with 1 positive genital PCR.

## DISCUSSION

This study is the first to examine the association of PCR-defined FGS with HIV-1 incidence. While barriers to implementation still exist, PCR for *Schistosoma* DNA detection in FGS diagnosis is reproducible, has high specificity, and can be performed on self-collected genital specimens [18]. We found that women with FGS were twice as likely to seroconvert than women in the comparison group, albeit with wide confidence intervals and no statistical evidence for a difference.

While some cross-sectional studies show an association between schistosomiasis and prevalent HIV-1 infection [13, 23], this association is not universally reported [34, 35]. The association between schistosomiasis and HIV-1 is complex and cross-study comparisons require consideration of many aspects, including schistosome species (*S. haematobium* vs *S. mansoni*), diagnostic tests used, assessment of genital involvement, and presentation of subgroup analyses, for example, by participant sex. Our findings, while limited by power, show a point estimate consistent with increased risk of incident HIV-1, but with a wide confidence interval. Recently, 2 case–control studies nested within longitudinal African cohorts have retrospectively assessed the association between schistosome infection status and HIV-1 seroconversion, with conflicting results [16, 36]. A Zambian study showed an increased risk of HIV-1 acquisition in *S. haematobium* antibody–positive women (adjusted hazard ratio, 1.4; *P *< .05), but not men [16]. Similar to our results, a study from Kenya and Uganda did not show an association between active schistosome infection and HIV-1 seroconversion, including in subgroup analyses by sex, schistosome species, and infection intensity [36]. Notably, however, neither of these nested case–control studies evaluated genital infection status. FGS may enhance HIV-1 vulnerability, with proposed mechanisms including cervicovaginal barrier dysfunction [37], local recruitment or activation of HIV-1 target cells [3], and *Schistosoma*-related alterations in integrin [38] or co-receptor [39] expression.

Schistosomiasis and FGS are preventable, and current World Health Organization control measures recommend praziquantel preventive chemotherapy [40]. However, current control programs do not universally achieve 75% coverage of school-aged children, representing substantial missed opportunities for prevention [41, 42]. The 2025 AIDS targets place communities at risk for HIV-1 in the center of societal, system, and service enablers with a call for between-sector integration and synergy to advance the HIV-1 response [43]. Programmatic synergy including integrated sexual and reproductive health programs could leverage and scale-up existing HIV-1 treatment and prevention resources to include FGS screening and treatment programs.

We carried out 2 ad hoc exploratory analyses. The intensity of schistosome infection, defined by serum CAA concentration, has been strongly correlated with HIV-1 prevalence [13]. Thus, first, we investigated whether *Schistosoma* DNA concentrations might be associated with HIV-1 infection in an analysis of 13 participants with FGS and moderate/high *Schistosoma* DNA concentrations. We found no evidence of an association between FGS and HIV-1 acquisition, albeit with wide confidence intervals. Additionally, participants with moderate- to high-intensity seminal egg excretion have higher seminal cytokine concentrations than *S. haematobium*–negative participants [44]. Thus, secondly, we investigated the association between multiple positive genital PCR specimens as a potential proxy marker of higher FGS burden and HIV-1 seroconversion in 13 women with ≥2 positive genital specimens for *Schistosoma* DNA. We found weak evidence of an association between FGS and HIV-1 acquisition, which was less pronounced in the age-adjusted estimates. These findings are hypothesis-generating for the association between FGS cervicovaginal disease burden or *Schistosoma* DNA concentrations and HIV-1.

This study was nested within a large population-based HIV-1 prevention trial and is the first prospective study to document FGS in Zambia, but it also had some relevant limitations. Similar to other FGS studies using imperfect available diagnostics, there is the risk of potential diagnostic misclassification, especially in this low-prevalence setting. We defined FGS by PCR positivity based on its semiquantitative nature and precedent in FGS diagnosis [17, 18, 45]. However, *S. haematobium* eggs in semen from a male sex partner could potentially be detected by PCR of vaginal specimens. We were unable to adjust for potential confounders beyond age and are thus unable to exclude unmeasured and residual confounding. This was related to the low number of HIV-1 seroconversions and FGS cases, which also resulted in a loss of power. Overall, the effect sizes suggest the possibility of a relationship we were not sufficiently powered to detect. While the prevalence of HIV-1 in the study population was high at 17.9%, the 2 participating communities were enrolled in HPTN 071 (PopART) as intervention sites, potentially reducing the number of HIV-1 seroconversions [25]. The prevalence of urinary *S. haematobium* infection in this study was 5.5% (27/492), lower than anticipated, defined by the World Health Organization as a low-prevalence area (<10% *S. haematobium* prevalence) [46]. Indeed, a 2013 survey done by the Zambian Ministry of Health reported a wide range of egg-patent prevalence in Livingstone, ranging from 3.3% to 73.3% (median, 15.0%; mean, 23.3%) in school-aged children, highlighting its focal distribution [47]. In addition, while schistosomiasis is endemic in all of Zambia’s 10 provinces [48] and can be found in urban locations, it is generally considered to be a focal disease of rural areas [49]. For all the above reasons, the presented estimates, obtained in a peri-urban setting, are subject to a high degree of imprecision and may not be generalizable to rural communities. Lastly, vaginal and cervical swabs were self-collected by participants, raising the potential for false-negative genital swabs. In future work, this could be addressed by measuring β-globin PCR as a positive control to confirm the presence of human DNA [50]. This study was developed based on a conceptual framework that describes a potentially causal relationship between FGS and HIV-1, with FGS as a potentially preventable and modifiable risk factor. In the literature, albeit in cross-sectional studies, there is evidence for biological plausibility [3, 8, 12] and large effect sizes for the association [7] between FGS and HIV-1. One of our study limitations was the temporality of HIV-1 and FGS diagnostics. HIV-1 seroconversion was measured in HPTN 071 (PopART) up to 3 years before participant enrollment in the study and subsequent FGS diagnosis. This sequencing assumes that FGS status and demographic descriptors at the time of genital PCR sampling are similar to those at the time of HPTN 071 (PopART) study entry and/or HIV-1 seroconversion. This assumption is reasonable given that FGS is thought to develop after childhood water exposure [33] and persist into adulthood with chronic genital lesions, often persisting despite treatment with praziquantel [51]. A large, prospective, longitudinal study in areas of higher *S. haematobium* endemicity is needed to evaluate incident HIV-1 infection in women with known *S. haematobium* and FGS status at study baseline. In future work, it will be important to continue to analyze HIV-1 outcomes by both *S. haematobium* infection status and FGS definition (PCR vs clinical findings) to evaluate HIV-1 risk profiles.

In conclusion, this analysis does not show evidence of association between FGS and HIV-1 incidence. The hypothesis-generating observations that FGS, and in particular higher FGS cervicovaginal disease burden or *Schistosoma* DNA concentrations, may be associated with HIV-1 acquisition should be investigated in a larger longitudinal study in a high–FGS prevalence area to better explore the role of FGS in HIV-1 acquisition.
